# Supplementary material for: Rare A360T Mutation Alters GSK3β(Ser9) Binding in the Cytosolic Loop of Presenilin 1, Influencing β-Catenin Nuclear Localization and Pro-Death Gene Expression in Alzheimer’s Disease Case
Source: Int J Mol Sci. 2023 Nov 30;24(23):16999. doi: 10.3390/ijms242316999 (PMC10707597; doi:10.3390/ijms242316999)
Supplement: Supplementary file 1 [file ijms-24-16999-s001.zip › ijms-2680242-Supplementary Materials.pdf]

Supplementary Materials:

SM File S1. A360T_HPO.yml. The script file executing for the Exomiser analysis in a mode including HPO terms.

SM File S2. A360T_noHPO.yml. The script file executing for the Exomiser analysis in a mode excluding HPO terms

SM Table SS1. List of 2321 differentially expressed genes in A360T cells versus normal controls (n=6) obtained using DESeq2 tool.

SM Table S2. List of 528 differentially expressed genes in A360T cells versus fEOAD cases (n=7) obtained using DESeq2 tool.

SM Table S3. List of 416 differentially expressed genes unique to A360T cells.

SM Table S4. Cell cycle and apoptosis-related genes unique to A360T cells.

SM Table S5. IPA in silico prediction based on DEGs transcriptomic results unique to A360T fibroblasts

SM Table S6. The list of human phenotype ontology (HPO) terms used by Exomiser.

SM Table S7. Variants called in A360T cases and non-mutated family member by Exomiser in a mode without HPO terms.

SM Table S8. The lists of SNPs selected by noHPO Exomiser mode (SM Table S7) and present in RNAseq DEGs dataset (SM Table S1), observed uniquely in the 5241 case (643 SNPs) and shared for 5241 and 7677 cases (19 SNPs).

SM Table S9. The IRF family found in WES and RNAseq data of the A360T patient.

SM Table S10. The list of biological terms and pathways (according to Reactome database 2022) selected based on noHPO mode Exomiser list generated for A360T cases.

SM Table S11. Following Exomiser analysis of WES data without defining HPO terms related to AD or neurodegeneration, we found The list of 42 genes with SNPs of significant score present in both 5241 and 7677 patient (A360T-carriers), and absent in 7676, a daughter of the 7677 case, the genes carried 64 SNPs.

SM Table S12. Functional in silico prediction for the 42 genes revealing a lists of altered pathways and processes, among of which there were top 18 were related to neurodegeneration in A360T carriers.

SM Table S13. The list of the process preselected by Pathview for the noHPO mode datasets preselected for the 5241, 7676, and 7677 cases.

SM Table S14. Variants called in A360T cases and non-mutated family members by Exomiser in a mode withHPO terms.

SM Table S15. The list of the 18 indels extracted from RNAseq dataset for the 5241 case base on the Exomiser analysis.

SM Figure S1. Subcellular localization of β-catenin, GSK3β and PS1 in A360T fibroblasts. PS1 was co-immunostained with β-catenin (A) or GSK3β(Ser9) (B) in A360T fibroblasts, other fEOAD-derived fibroblasts, and control ones. Confocal images are presented as split channels in green for presenilin 1, and in red for β-catenin (A) or GSK3β(Ser9) (B). Merged images are on the right.

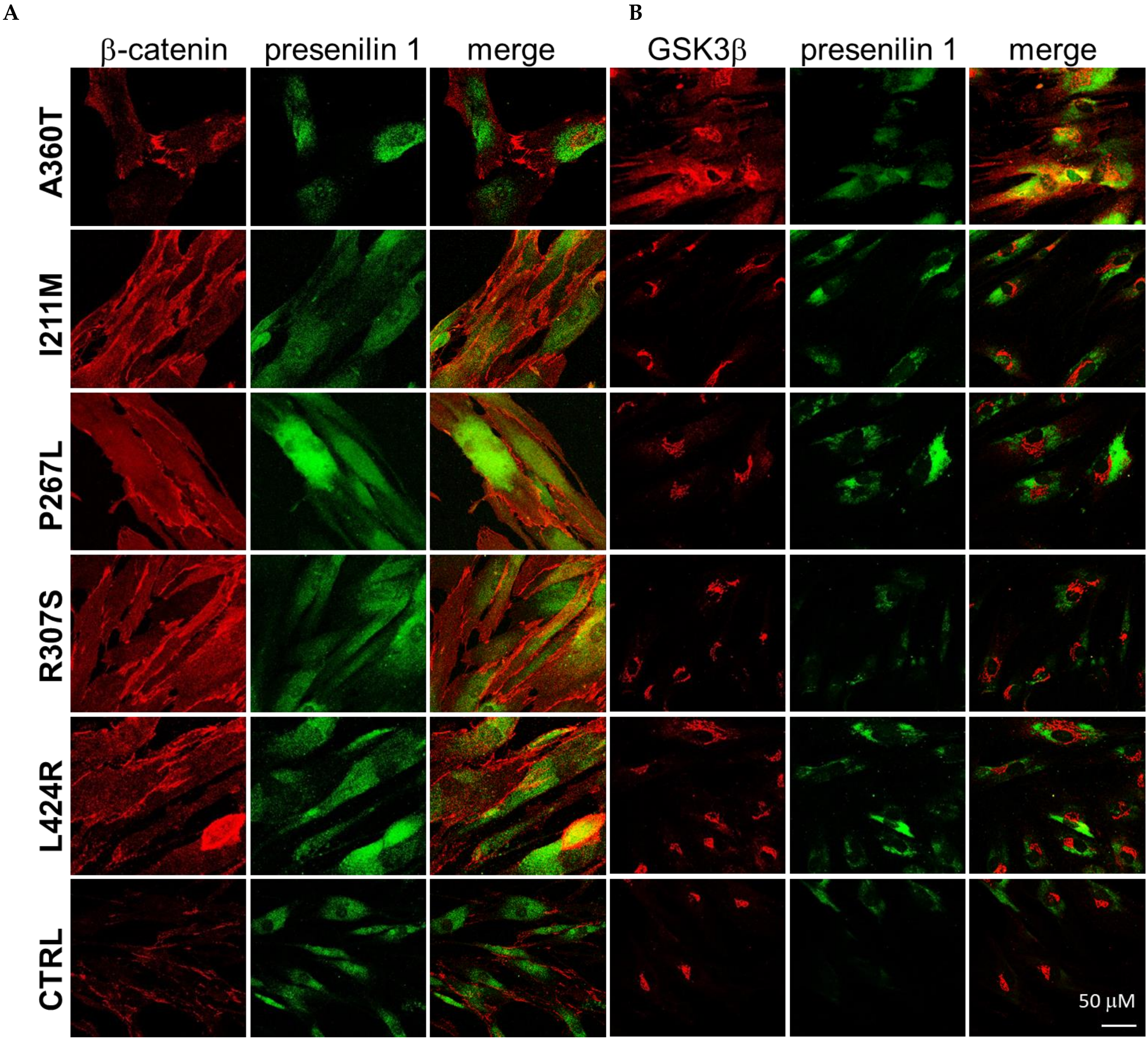

SM Figure S2. Fluorescence intensity of GSK3b and PS1. Fluorescence intensity was determined and expressed as corrected total cell fluorescence (CTCF), which equals to integrated density, minus the area of the selected cell, multiplied by the mean fluorescence of background readings (https://theolb.readthedocs.io/en/latest/imaging/measuring-cell-fluorescence-using-imagej.html). CTCF was determined for GSK3b (A) and PS1 (B) separately, and the ratio of the two values was calculated (C) (n=6).

(**A**)

(**B**)

(**C**)

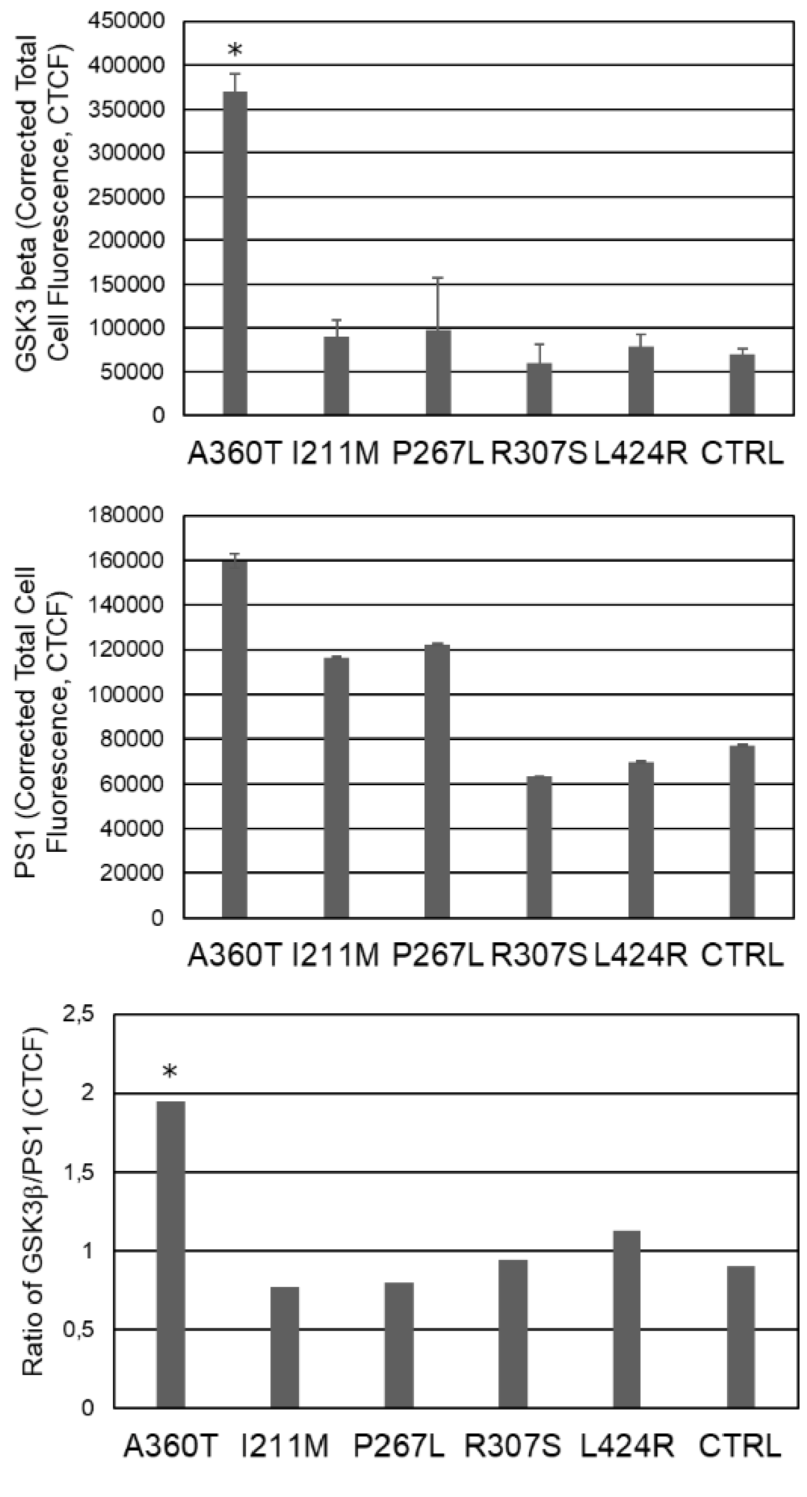

SM Figure S3. Subcellular nuclear localization of β-catenin, GSK3β, and PS1 in A360T fibroblasts. The images were collected using fluorescence microscopy and represent all the channels separated as black-white images and merged images (GSK3b in red, PS1 in green, and nuclei in blue).

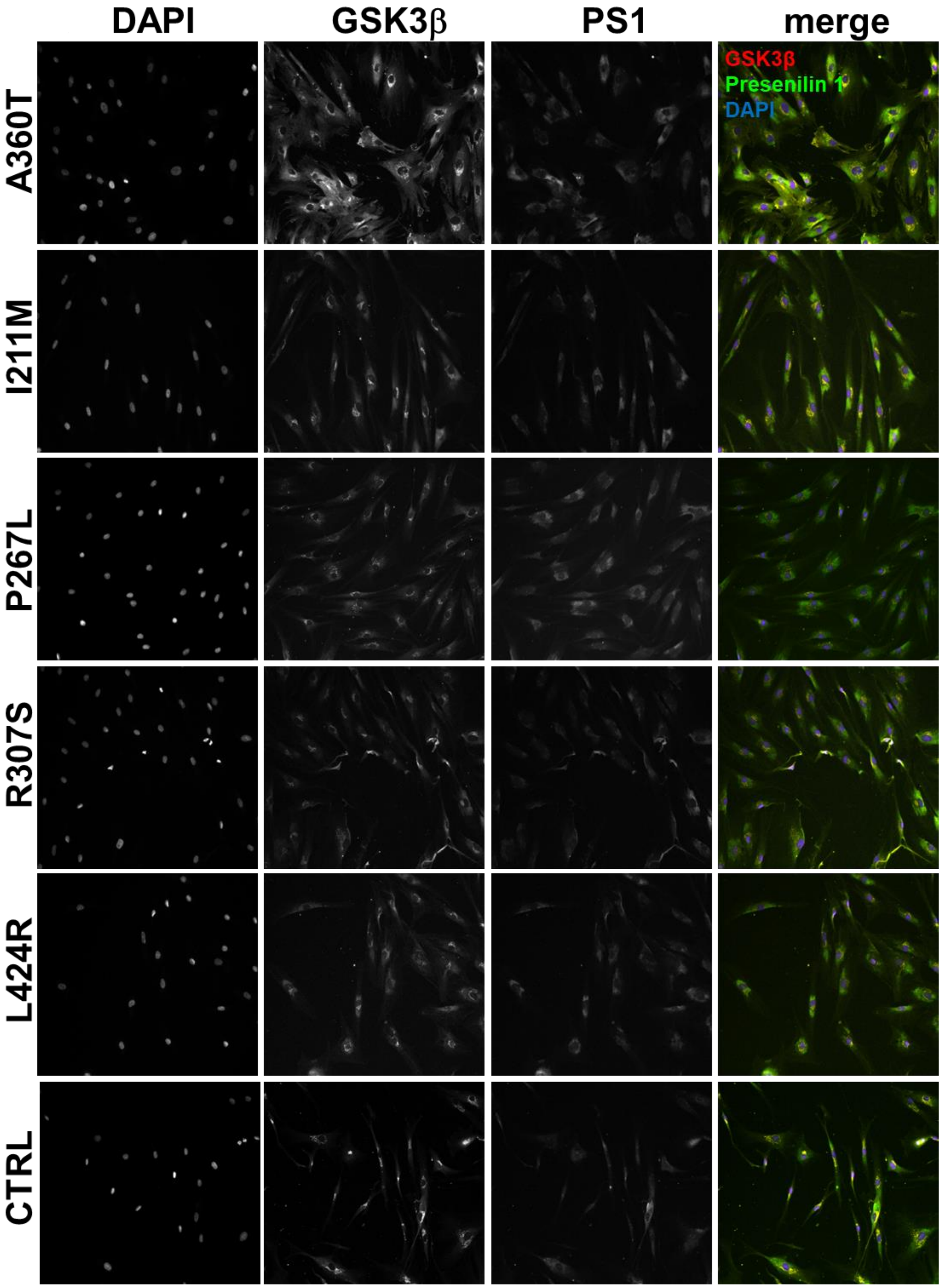

SM Figure S4. Amyloid produced by A360T fibroblasts, and those from other fEOAD patients and healthy controls. The concentration of amyloid-beta 1-42 and amyloid-beta 1-40 was determined in cell culture medium collected from fibroblasts using ELISA assays (Millipore, cat no. EZHS42 and cat no. EZHS40), n = 3, * $p < 0.05$.

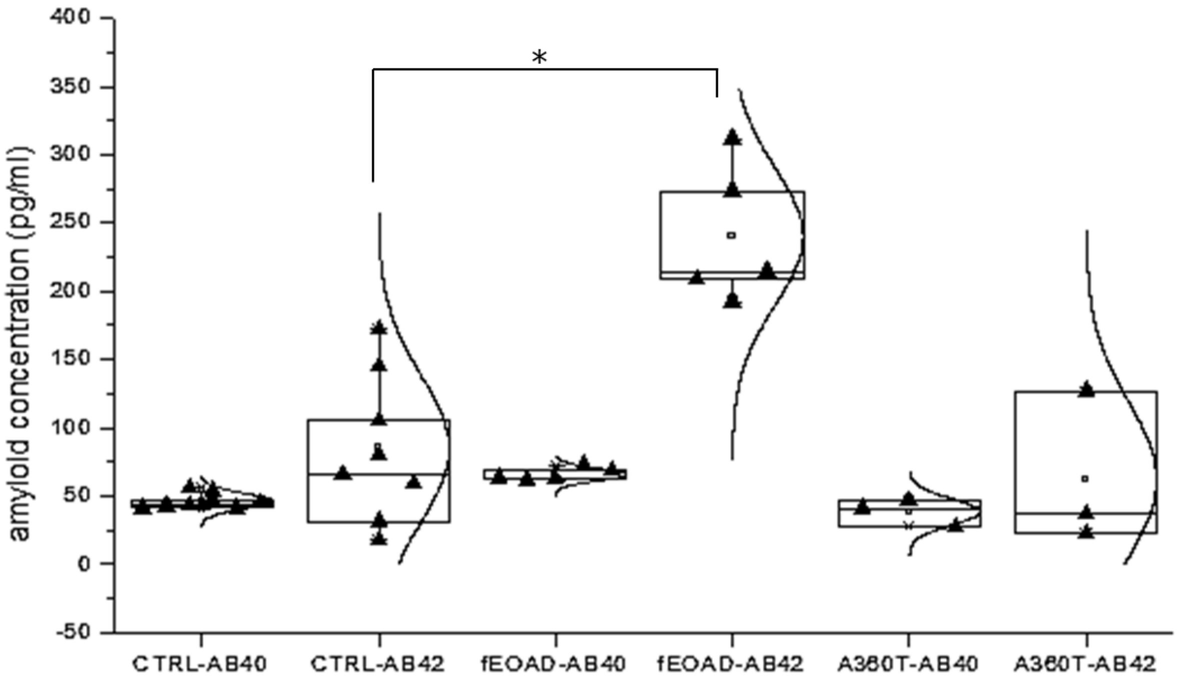

SM Figure S5. Cell cycle and apoptosis-related pathways generated upon Pathview and GAGE analysis based on the lists of genes diferentailly expressed uniquely to A360T cells.

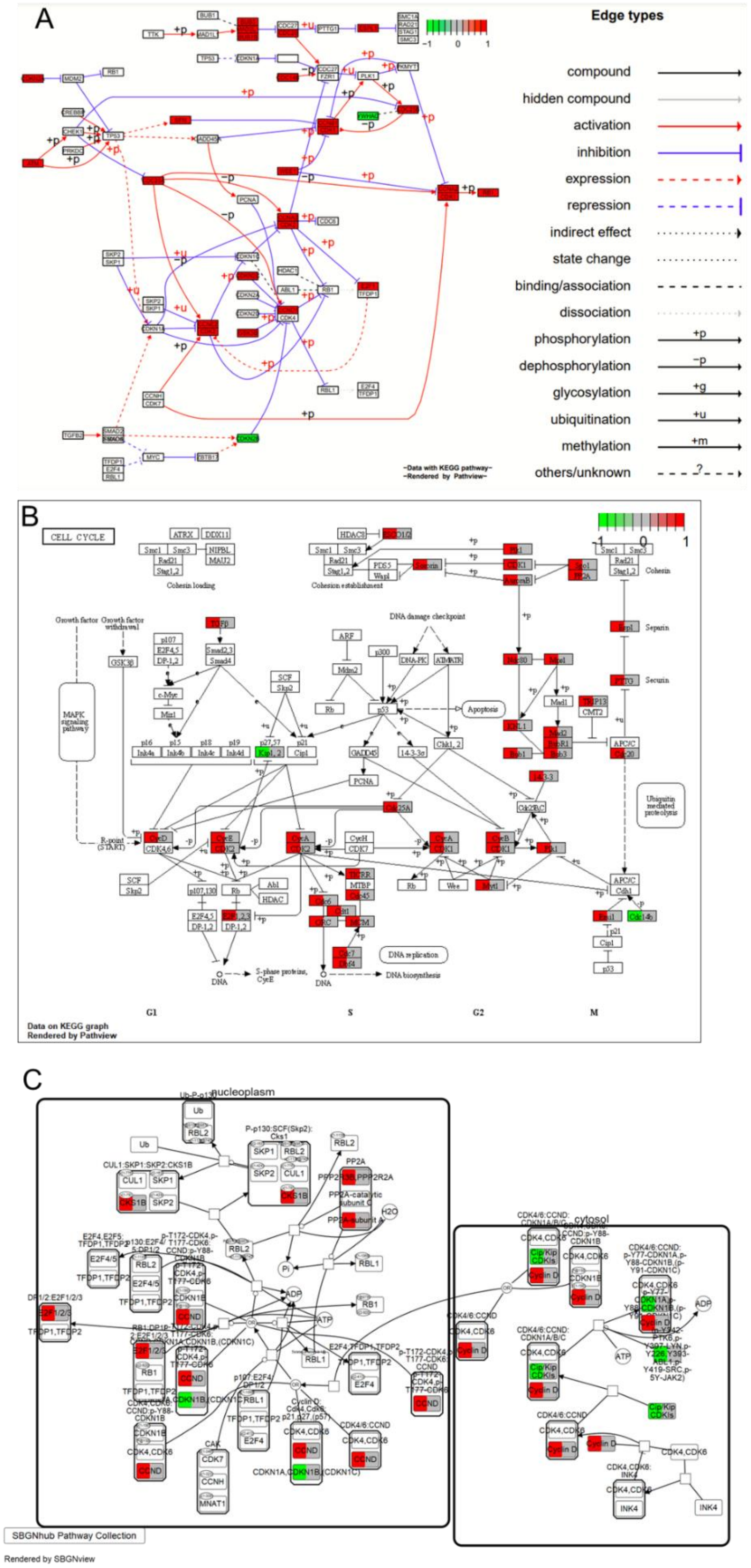

Supplementary description of the fEOAD patients (other than A360T cases).

R307S patient. Male patient,54 years old at the onset with the mixed clinical phenotype of Alzheimer's disease and frontotemporal dementia (FTD). In family history, a case of brother diagnosed with the clinical phenotype of rapidly progressive FTD, reported age of death 55 years, but no data are available about the genetic background of FTD patient's brother. At age 54 patient started to display difficulties in maintenance of concentration, orientation, and in performing work (intellectual academic work). At age 56 the patient was hospitalized with recognized mild cognitive impairment mainly affecting executive functions, MMSE scores 29, magnetic resonance imaging (MRI) revealed cortical frontotemporal-parietal atrophy. At ages 57 and 58 the dementia was progressing. At age 59 the patient was diagnosed with severe dementia and needed total care, the MMSE result was 7. At the final stage of the disease at age 60 the patient presented mutism, and remained immobile, periodically excited, with abnormal fluid and solid food intake. Age of death: 63 years.

P267L patient. Female, with a high-school education, working as a clerk. The first symptoms, mostly in attention and emotional liability were noticed at age 47. Due to positive family history, the proband was admitted to an outpatient clinic. Medical history revealed hypertension for 25 years, and anxiety disorders. Based on the CT (normal), as well as the neurological (normal), psychiatric (anxiety disorders), and neuropsychological examinations, the diagnosis of Mild Cognitive impairment (MCI; Clinical Dementia Rating (CDR) scored as 0.5) (Morris, 1993) was set at age 48. In November 2013, the patient was hospitalized to perform a lumbar puncture, which indicated a pattern for AD (decreased level of Aβ, and increased levels of total tau and phospho-tau); computed tomography (CT) was normal, with no changes in mood and cognition, the neuropsychological assessment showed improvement in comparison to the previous examination (CDR 0). In 2014, the patient was informed about the results of the genetic testing and biomarkers results and her mood significantly worsened into severe depression. A neuropsychological assessment revealed a general worsening of cognitive functioning, with deficits mostly pronounced in executive, language, and memory domains. She remained independent in everyday activities (CDR 0.5). In 2015, her cognition became more disturbed and the patient claimed problems with spatial orientation and visual-spatial functions. She resigned from her job due to emotional problems. There was also a decline in the ability to perform complex everyday tasks, but she remained independent (CDR 0.5). The most recent assessment (in 2017) revealed worsening both in cognitive functioning (disturbed episodic memory, worsening in visuospatial and attention domains) as well as in everyday tasks and the patient requires partial supervision. Aβ42 CSF level of the patient was 455.42 pg/mL (healthy threshold:609.54 pg/mL) indicating Aβpathology, total tau protein was 752.1 pg/mL (healthy threshold:277.02 pg/mL) and phosphorylated tau CSF level was 100.7 pg/mL (healthy threshold:55.08 pg/mL), indicating tau pathology.

I211M patient. Female, 67 years old at onset with posterior cortical atrophy (PCA) with symptoms of cortical visual dysfunction, associated with AD type amyloid plaques and neurofibrillary tangles. 48-year-old mutation carrying the son of the patient stayed completely cognitively healthy. Aβ42 CSF level of the patient was 384.6 pg/mL (healthy threshold is 609.54 pg/mL, indicating amyloid AD pathology), total tau protein was 378.9 pg/mL healthy threshold is 277.02 pg/mL, indicating tau AD pathology) and phosphorylated tau CSF level was 84.2 pg/mL (healthy threshold is 55.08 pg/mL, indicating tau AD pathology).

L153V patient. Female patient with first symptoms at age 34 years with MMSE=24 and overall good health condition. Diagnosed with familial AD at age 39 with MMSE=20 and CDR=-2 (in 2013) and characterized by rapidly increasing cognitive disability within 6 months, with prominent impairment of episodic memory and accompanied by behavioural and psychological symptoms of dementia (BPSD); strong mood swings and anxiety. Patient's detailed neuropsychological examination revealed no deficits in language, problem-solving, thinking and speed. However, deficits were observed in divided and sustained attention, learning ability, immediate and delayed memory, with remark distractibility. Executive functions were severely impaired, with problems in planning and monitoring. Visuo-spatial functions were slightly impaired due to problems in perception. Electro-encephalographic (EEG) examination (2013.04) revealed episodic changes in fronto-temporal vicinity of free theta waves, against the normal function. CT scan of the head revealed moderate cortico-subcortical atrophy of the brain. The patient was subjected to treatment with donepezil 10mg from April 2013. The same mutation was identified in the patient's brother, diagnosed with fEOAD at age 44, progressing rapidly within the next 4 years (died in 2007). Patient's second brother is currently diagnosed with severe dementia and taken under medical care in another hospital in Poland (Cracow).

L424R brother 1 patient. 36 years old at onset, with extremely rapid disease progression within 2 years, with increasing cognitive impairment. Patient with a strong familial history (patients father, brother, sister, uncle, and uncle's sons affected, carrying the mutation), as demonstrated at the pedigree. Neurological examination revealed features of extrapyramidal syndrome with predominant tremor. Neuropsychological examination revealed several disturbances described below. Obtained results pointed to severe cognitive slowing, major problems in divided and sustained attention, learning of new information, delayed memory, with prominent distractibility but only discrete impairment of immediate memory. Language problems were mostly present in poor fluency and comprehension difficulties. Visuo-spatial dysfunctions were present in perception as well as in constructional praxis. Executive functions were also impaired, with problems in flexibility, planning and monitoring with concrete thinking. MMSE score was 20. CT scan of the head performed at age 35 revealed moderate cortical atrophy of the brain, but overall CT scans of the cortex and subcortical structures, as well as the ventricular system image, were correct and no lesions in the CNS were found. EEG revealed episodic changes in the fronto-temporal lobe in the form of numerous symmetric and synchronous slow theta waves with low voltage background of basal activity. The patient was treated with donepezil.

L424R brother 2 patient. 31 years old at the onset with FAD diagnosis, first symptoms manifested as MCI had started at age 26, with rapidly progressing memory loss. Patient with a strong familial history, as described above (brother of the patient described above). In the neuropsychological assessment, no problems in language and speed were observed, however, deficits in divided and sustained attention, learning, immediate and delayed memory, with distractibility were present. Executive functions were severely impaired, with problems in planning and monitoring, inhibition, impulsiveness, and the lack of mental flexibility, abstraction and insight were present. Visuo-spatial functions were declined due to perceptual deficits. MMSE result was 22. CT scan of the head performed at age 31 revealed overall correct pictures of the cortex and subcortical structures. The ventricular system image was correct and no lesions in the CNS were found. The patient was subjected to a treatment with donepezil.
