# Supplementary material for: Rare A360T Mutation Alters GSK3β(Ser9) Binding in the Cytosolic Loop of Presenilin 1, Influencing β-Catenin Nuclear Localization and Pro-Death Gene Expression in Alzheimer’s Disease Case
Source: Int J Mol Sci. 2023 Nov 30;24(23):16999. doi: 10.3390/ijms242316999 (PMC10707597; doi:10.3390/ijms242316999)
Supplement: Supplementary file 1 [file ijms-24-16999-s001.zip › SM Table S9. The IRF family found in the A360T patient.pdf]

SM Table S9. The IRF family found in the A360T patient.

Gene ID	log_2 Fold Change	P-value	Description
IRF1	-0.667	0.786	interferon regulatory factor 1 [HGNC:6116]
IRF2	-1.339	0.003	interferon regulatory factor 2 [HGNC:6117]
IRF2BP1	-0.682	0.400	interferon regulatory factor 2 binding protein 1 [HGNC:21728]
IRF2BP2	-0.872	0.0901	interferon regulatory factor 2 binding protein 2 [HGNC:21729]
IRF2BPL	-0.895	0.304	interferon regulatory factor 2 binding protein-like [HGNC:14282]
IRF3	-0.401	0.872	interferon regulatory factor 3 [HGNC:6118]
IRF4	-2.742	0.245	interferon regulatory factor 4 [HGNC:6119]
IRF5	-0.373	0.496	interferon regulatory factor 5 [HGNC:6120]
IRF6	-2.458	0.292	interferon regulatory factor 6 [HGNC:6121]
IRF7	-0.667	0.681	interferon regulatory factor 7 [HGNC:6122]
IRF9	-0.847	0.943	interferon regulatory factor 9 [HGNC:6131]
